# Structural Insights Uncover the Specific Phosphoinositide Recognition by the PH1 Domain of Arap3

**DOI:** 10.3390/ijms24021125

**Published:** 2023-01-06

**Authors:** Youjia Zhang, Liang Ge, Li Xu, Yongrui Liu, Jiarong Wang, Chongxu Liu, Hongxin Zhao, Lei Xing, Junfeng Wang, Bo Wu

**Affiliations:** 1 High Magnetic Field Laboratory, Key Laboratory of High Magnetic Field and Ion Beam Physical Biology, Hefei Institutes of Physical Science, Chinese Academy of Sciences, Hefei 230031, China; 2 Science Island Branch of Graduate School, University of Science and Technology of China, Hefei 230026, China; 3 Institute of Biotechnology and Health, Beijing Academy of Science and Technology, Beijing 100089, China; 4 School of Life Sciences, University of Science and Technology of China, Hefei 230026, China; 5 Institute of Physical Science and Information Technology, Anhui University, Hefei 230601, China

**Keywords:** Arap3, PH domain, complex structure, PI(3,4,5)P3, PI(4,5)P2, NMR, cancer cell invasion

## Abstract

Arap3, a dual GTPase-activating protein (GAP) for the small GTPases Arf6 and RhoA, plays key roles in regulating a wide range of biological processes, including cancer cell invasion and metastasis. It is known that Arap3 is a PI3K effector that can bind directly to PI(3,4,5)P3, and the PI(3,4,5)P3-mediated plasma membrane recruitment is crucial for its function. However, the molecular mechanism of how the protein recognizes PI(3,4,5)P3 remains unclear. Here, using liposome pull-down and surface plasmon resonance (SPR) analysis, we found that the N-terminal first pleckstrin homology (PH) domain (Arap3-PH1) can interact with PI(3,4,5)P3 and, with lower affinity, with PI(4,5)P2. To understand how Arap3-PH1 and phosphoinositide (PIP) lipids interact, we solved the crystal structure of the Arap3-PH1 in the apo form and complex with diC4-PI(3,4,5)P3. We also characterized the interactions of Arap3-PH1 with diC4-PI(3,4,5)P3 and diC4-PI(4,5)P2 in solution by nuclear magnetic resonance (NMR) spectroscopy. Furthermore, we found overexpression of Arap3 could inhibit breast cancer cell invasion in vitro, and the PIPs-binding ability of the PH1 domain is essential for this function.

## 1. Introduction

Recruitment of peripheral membrane proteins to the plasma membrane (PM) is a critical step in many cell signaling pathways [1,2]. The process can be driven by interaction of the target protein with specific lipid in PM, including some phosphoinositides (PIPs) such as phosphatidylinositol 3,4,5-trisphosphate [PI(3,4,5)P3] [3,4,5]. PI(3,4,5)P3 is generated from phosphatidylinositol 4,5-bisphosphate [PI(4,5)P2] by Class I phosphoinositide 3-kinase (PI3K) [6]. It serves as a phospholipid second messenger to recruit and activate numerous PI3K effectors at PM, and is involved in a wide variety of biological processes [7,8,9,10].

**Figure 1:**
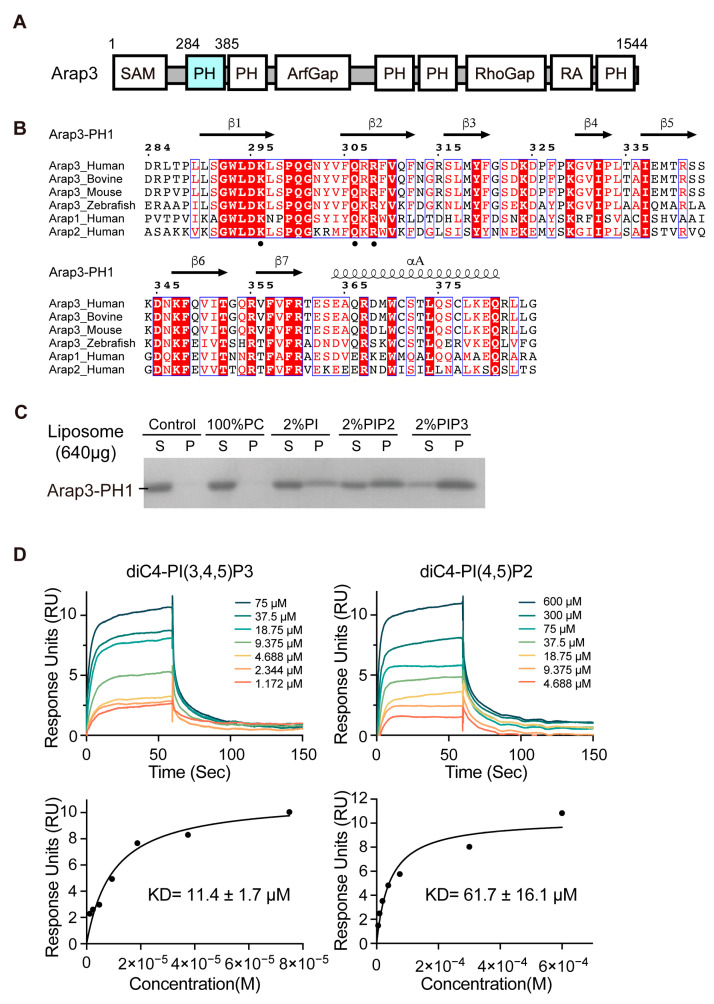
Phospholipid-binding abilities of Arap3-PH1 domain analyzed using liposome pull-down assay and SPR measurements. (**A**) Schematic representation of human Arap3 protein. The first PH domain of Arap3 is marked in cyan. (**B**) Sequence alignment of Arap3-PH1 orthologs in vertebrates and human Arap1-PH1, Arap2-PH1. Sequence accession number in the Uniprot database are: human, Q8WWN8; bovine, E1BBA0; mouse, Q8R5G7; zebrafish, A0A140LH27; human Arap1, Q96P48; human Arap2, Q8WZ64. Alignment was performed using Clustal X and illustrated with ESPript 3.0. Strictly conserved (white letters filled with red color) and conservatively substituted (red letters with blue box) residues are denoted. The secondary structure element for human Arap3-PH1 is labeled on the top. The KXnQXR motif are marked by black dots. (**C**) Arap3-PH1 (20 μg) mixed with liposomes (640 μg) composed of 98% PC as the fixed component and 2% of specific phospholipids, respectively. Proteins in the absence of liposome were used as a control. After centrifugation, the pellet (P) and supernatant (S) were analyzed by SDS/PAGE and Coomassie. (**D**) SPR measurements of the binding affinities of the Arap3-PH1 domain for diC4-PI(3,4,5)P3 and diC4-PI(4,5)P2. The upper panel shows representative sensorgrams of diC4-PI(3,4,5)P3 (left) and diC4-PI(4,5)P2 (right) when mixed with Arap3-PH1. Data were collected by injecting increasing concentrations of diC4-PI(3,4,5)P3 and diC4-PI(4,5)P2 samples over Arap3-PH1 proteins immobilized on the surface of a CM5 biochip. The lower panel shows representative binding curves fitting for diC4-PI(3,4,5)P3 (left) and diC4-PI(4,5)P2 (right) during their interaction with Arap3-PH1. A one-site binding model was utilized to fit the curves. The experiment was carried out in triplicate. The KD value is presented as mean ± SD, n = 3.

Arap3 is a PI3K effector protein that was originally identified from a screen for PI(3,4,5)P3-binding proteins [11]. It belongs to the ARAP family and functions as a dual GTPase-activating protein (GAP) for the small GTPases Arf6 and RhoA [12]. Arap3 structurally consists of an N-terminal sterile alpha motif (SAM) domain, two catalytic GAP domains, ArfGAP and RhoGAP, a Ras-associating (RA) domain and five pleckstrin homology (PH) domains (Figure 1A). It is uncommon for a protein to contain 5 PH domains. Arap3 plays essential roles in diverse biological processes, including cell adhesion and migration [13,14,15], developmental angiogenesis [16], and lymphangiogenesis [17]. It is also involved in several cancers. It inhibits peritoneal dissemination of scirrhous gastric carcinoma cells [18]. It interacts directly with NEDD9, which is an established marker of invasive and metastatic cancers, and is involved in regulating mesenchymal cell invasion and metastasis of breast cancer [19]. Moreover, the expression level of ARAP3 might be a useful indicator of the metastatic likelihood of the basal-like breast tumors [20].

PI(3,4,5)P3 binding is essential for Arap3 function, which drives recruitment of Arap3 to the plasma membrane to facilitate interaction with its GTPase substrates [11,21]. Despites the critical roles of binding to PI(3,4,5)P3, how Arap3 interacts with PI(3,4,5)P3 is still elusive. It is well established that the N-terminal first pleckstrin homology (PH1) domain is essential for Arap3 to bind PI(3,4,5)P3. Mutagenesis of two conserved arginine residues (R307A, R308A) within the PH1 domain renders full-length Arap3 unable to bind PI(3,4,5)P3 in vitro, and thus prevents its recruitment to plasma membrane in vivo [11]. This Arap3 mutant leads to embryonic lethality [16,22]. Unexpectedly, a recent study employing a competitive PI(3,4,5)P3-conjugated bead binding assay and cell lysates expressing different Arap3 fragments led to a conclusion that neither the PH1 nor the other four PH domains of Arap3 are sufficient to bind PI(3,4,5)P3 in isolation; rather, a fragment comprising two PH domains and an N-terminal linker is minimally required for binding [23]. The revealed PI(3,4,5)P3-binding mechanism of Arap3 seems to be very unusual [24,25,26].

In this study, we first expressed and purified the recombinant Arap3-PH1 domain and examined its lipid binding ability using liposome pull-down assay and surface plasmon resonance (SPR). Contrary to previous studies [23], we found that Arap3-PH1 alone is capable of binding PI(3,4,5)P3 and also PI(4,5)P2, albeit with lower affinity. To understand how Arap3-PH1 and PIP lipids interact, we solved the crystal structure of the Arap3-PH1 in its free state and in a complex with diC4-PI(3,4,5)P3, a soluble analog of PI(3,4,5)P3. Moreover, we have characterized the interactions of Arap3-PH1 with diC4-PI(3,4,5)P3 and diC4-PI(4,5)P2 by NMR. In addition, we found that a cancer-associate point mutation within the Arap3-PH1 domain (R308H) abolishes its binding to PI(3,4,5)P3 lipid, and impairs the capacity of Arap3 to inhibit breast cancer cell invasion in vitro.

## 2. Results

### 2.1. The PH1 Domain of Arap3 Is Sufficient to Bind PIPs and Prefers PI(3,4,5)P3

Previous experiments, based on PI(3,4,5)P3-conjugated bead binding assay using cell lysates, have shown that the PH1 domain is essential for Arap3 to bind PI(3,4,5)P3 [11], but it alone cannot bind PI(3,4,5)P3 [23]. However, sequence analyses show that Arap3-PH1 contains a KXnQXR motif in the β1-β2 region (Figure 1B), which is identical to the canonical KXn(K/R)XR motif in PIPs binding-PH domains [27], except for the substitution of (K/R) residue by Gln (Q306) in Arap3-PH1. Such a Gln residue substitution at this position can also been found in a few PIPs-binding PH domains, such as the PH domain of P-Rex1 [28]. The sequence analysis led us to ask whether the Arap3-PH1 alone is sufficient to bind PI(3,4,5)P3 and/or other PIPs.

To test this hypothesis, we expressed and purified the Arap3-PH1 protein (residues 284–385), and then examined its PIPs binding ability using a liposome pull-down assay, a widely used direct detection method for protein-lipid interactions in vitro. We used phosphatidylcholine (PC), one of the major phospholipids in PM, as the main composition of liposomes, and 100% PC-liposomes were used as a negative control. PI(3,4,5)P3-containing liposomes were prepared by incorporating 2% PI(3,4,5)P3 into the PC-liposomes. In addition, to explore the PIPs binding specificity of Arap3-PH1, we also made another two distinct liposomes, PI(4,5)P2-containing and phosphatidylinositol (PI)-containing liposomes. In this assay, the purified Arap3-PH1 protein was pre-incubated with liposomes. After ultracentrifugation, the supernatant and pellets were analyzed by SDS–PAGE. As shown in Figure 1C, Arap3-PH1 bound efficiently and preferentially to PI(3,4,5)P3 and exhibited decreased binding to PI(4,5)P2, whereas no detected binding to PC and a much weaker binding to PI were observed. To further examine the interaction of Arap3-PH1 domain with PIPs head groups, we performed surface plasmon resonance (SPR) analysis using diC4-PI(3,4,5)P3 and diC4-PI(4,5)P2, the water-soluble lipid analogs of PI(3,4,5)P3 and PI(4,5)P2, respectively. As a result, diC4-PI(3,4,5)P3 exhibits a 5.4-fold stronger binding (KD ≈ 11.4 μM) to Arap3-PH1 than diC4-PI(4,5)P2 (KD ≈ 61.7 μM) (Figure 1D).

Together, in contrast to a previous study [23], our data clearly demonstrated that Arap3-PH1 alone is sufficient to bind PIPs and it prefers PI(3,4,5)P3 for binding, which is consistent with the reported in-cell data that full-length Arap3 is a PI(3,4,5)P3 binding protein [11].

### 2.2. Crystal Structures of Unliganded and diC4-PI(3,4,5)P3-Bound Arap3-PH1 Domain

**Figure 2:**
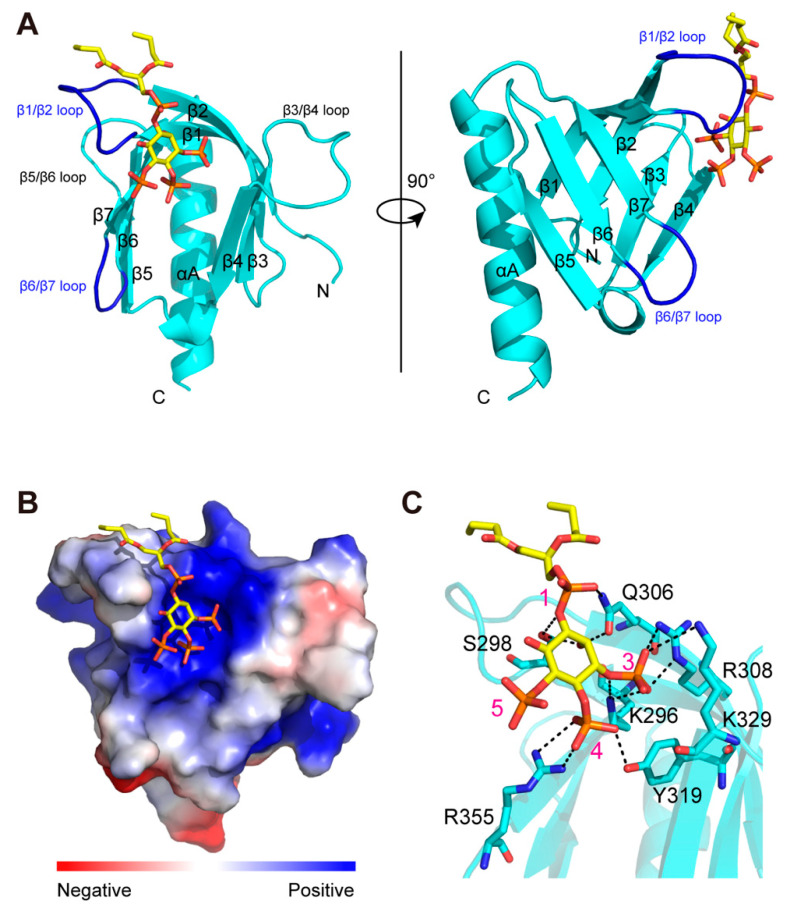
Structure of the Arap3-PH1 domain in complex with diC4-PI(3,4,5)P3. (**A**) Cartoon diagram of Arap3-PH1 complexed with diC4-PI(3,4,5)P3. Arap3-PH1 is colored turquoise, with secondary structures labeled. The loops of β1/β2 and β6/β7 that interact directly with diC4-PI(3,4,5)P3 are colored blue. The diC4-PI(3,4,5)P3 (gold) is shown in stick mode. (**B**) Surface electrostatic potential of Arap3-PH1 complexed with diC4-PI(3,4,5)P3. Blue areas, positive; red areas, negative. (**C**) Detailed interactions of the diC4-PI(3,4,5)P3 with Arap3-PH1 domain. The side chains of crucial residues are shown in stick mode and labeled, respectively. The phosphate groups on diC4-PI(3,4,5)P3 are also labeled. Selected hydrogen bonds or salt bridges are shown as dotted lines.

To understand how Arap3-PH1 domain recognizes PI(3,4,5)P3, we determined the crystal structures of unliganded and diC4-PI(3,4,5)P3-bound Arap3-PH1 domain, which refined to 2.1 and 3.3Å resolution, respectively (Table 1). Arap3-PH1 domain adopts a canonical PH domain fold, which consists of a seven-stranded β-barrel that is capped at one end by a long C-terminal α-helix (Figure 2A). The other end of the β-barrel is open and features three loops (β1/β2, β3/β4 and β6/β7), which are hypervariable in both length and sequence in presently known PH domain structures. The overall structures of Arap3-PH1 in free and in complex are almost identical, with an overall Ca RMSD of 0.85 Å. However, a significant structural change upon diC4-PI(3,4,5)P3 binding is observed in the region of β1/β2 loop (S298-V304), which exhibits an outward movement, with a Ca RMSD of 1.52 Å (Figure S1).

In the complex, the diC4-PI(3,4,5)P3 binds within a highly positively charged pocket on the open end of the β-barrel, which corresponds to the canonical phosphoinositide-binding site (Figure 2B). The 1-phosphate group of diC4-PI(3,4,5)P3 forms hydrogen bonds with the side chains of S298 in β1/β2 loop and Q306 in β2 (Figure 2C). The 3-phosphate group interacts with the side chains of K296 in β1, R308 in β2 and K329 in β4. The 4-phosphate group forms a hydrogen bond with the side chains of Y319 in β3. It also interacts with R355 in β6/β7 loop. It is interesting to note that the 5-phosphate is orientated toward the solvent and does not make hydrogen bonds with the protein.

The revealed PI(3,4,5)P3 head group recognition mechanism of Arap3-PH1 is quite similar to that of the P-Rex1 PH domain [28], although the sequence identity is rather low (∼13.68%). As with Arap3-PH1, P-Rex PH domain also preferentially binds PI(3,4,5)P3. As shown in Figure S2, all the seven key residues (K296, S298, Q306, R308, Y319, K329, R355) in Arap3-PH1 that interact directly with the PI(3,4,5)P3 head group can also be seen in the corresponding site of P-Rex1 PH domain, only with the substitutions of K329 for Arg, and R355 for Lys.

### 2.3. NMR Characterize of Arap3-PH1 Domain Binding to PI(3,4,5)P3 and PI(4,5)P2 Head Groups in Solution

To further examine the interaction of the Arap3-PH1 domain with PIPs head groups in solution, we subsequently performed NMR experiments with diC4-PI(3,4,5)P3 and diC4-PI(4,5)P2. Assignment of the Arap3-PH1 signals was made by performing a series of triple resonance NMR experiments using ^15^N, ^13^C-labeled Arap3-PH1. In total, 80 backbone ^1^H, ^13^C, and ^15^N NMR assignments out of 97 non-proline residues were obtained unambiguously (Figure S3). Some resonance signals of the backbone amide groups could not be observed in ^1^H–^15^N HSQC spectra, such as the β6/β7 loop residues.

**Figure 3:**
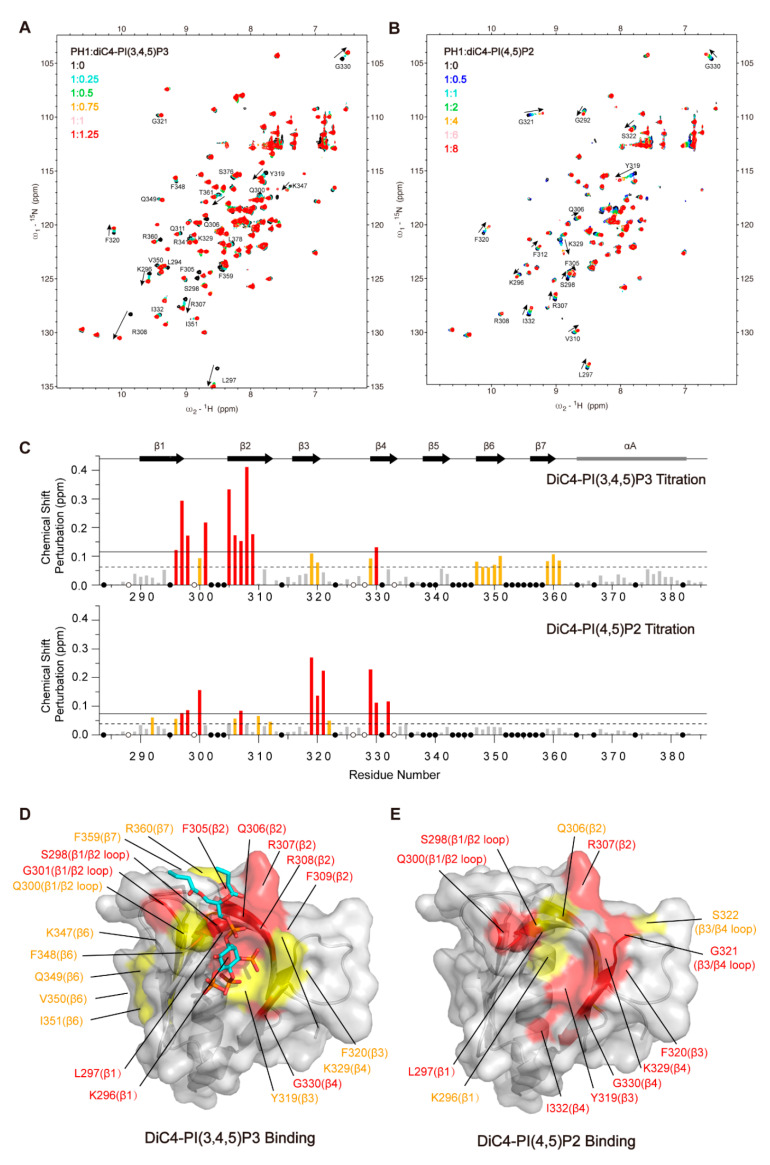
The binding interfaces of Arap3-PH1 for diC4-PI(3,4,5)P3 and diC4-PI(4,5)P2 revealed by NMR titration. (**A**) Overlay of ^1^H-^15^N HSQC spectra of Arap3-PH1 in the absence (black) and in the increasing amounts of diC4-PI(3,4,5)P3. The molar ratios of the protein to diC4-PI(3,4,5)P3 are shown in the inset: 1:0 (black), 1:0.25 (turquoise), 1:0.5 (lime green), 1:0.75 (orange), 1:1 (pink) and 1:1.25 (red). (**B**) Overlay of ^1^H-^15^N HSQC spectra of Arap3-PH1 in the absence (black) and in increasing amounts of diC4-PI(4,5)P2. The molar ratios of the protein to diC4-PI(4,5)P2 are shown in the inset: 1:0 (black), 1:0.5 (royal blue), 1:1 (turquoise), 1:2 (lime green), 1:4 (orange), 1:6 (pink) and 1:8 (red). (**C**) The chemical shift perturbations (CSPs) of each residue during NMR titrations (up, diC4-PI(3,4,5)P3 titration; down, diC4-PI(4,5)P2 titration) are calculated and shown with the secondary elements on top. White dots indicate pro residues. Black dots indicate residues with no data. The mean value and the mean value plus one standard deviation are indicated by dash and solid lines, respectively. Residues with CSPs between mean value and mean value plus one standard deviation are colored gold, and above mean value plus one standard deviations are colored red. (**D**,**E**) Surface representations of the Arap3-PH1 structure with the perturbed residues upon binding to diC4-PI(3,4,5)P3 and diC4-PI(4,5)P2 are colored and labeled.

The addition of soluble diC4-PI(3,4,5)P3 into ^15^N-labeled Arap3-PH1 resulted in specific ^1^H-^15^N chemical-shift perturbations (CSP) (Figure 3A,C). The most perturbed residues (with CSPs above mean value plus one standard deviation) are clustered mainly in the PI(3,4,5)P3-binding pocket, including β1 (K296-L297), β2 (F305-F309) and β1/β2 loop (S298 and G301) regions of the Arap3-PH1 domain (Figure 3D), which is highly consistent with the complex crystal structure. In addition, other regions, including β6 (K347-I351) and the C-terminal of β7 and its flanking residues (F359, R360 and S361), were also perturbed (with CSPs above mean value). Those regions do not interact with diC4-PI(3,4,5)P3 directly, and perturbation in these regions may be due to conformation change upon diC4-PI(3,4,5)P3 binding.

Titration of diC4-PI(4,5)P2 into a ^15^N-labeled Arap3-PH1 also led to significant CSPs for a number of residues (Figure 3B,C). When mapped onto the structure, these residues were also located in the positively-charged binding pocket and its surrounding regions, indicating the binding site for diC4-PI(4,5)P2 (Figure 3E). However, distinct differences in CSPs pattern were observed when compared with diC4-PI(3,4,5)P3 binding. As shown in Figure 3C,E, diC4-PI(4,5)P2 induced much smaller changes in the regions of β1 and β2, but larger changes in the regions of β3 (Y319 and F320), β3/β4 loop (G321) and β4 (K329 and I332) compared with diC4-PI(3,4,5)P3. In addition, unlike PI(3,4,5)P3, changes in β6 and β7 were not observed upon PI(4,5)P2 binding. The NMR data suggested that diC4-PI(4,5)P2 may bind more peripherally than diC4-PI(3,4,5)P3, which is in agreement with the observation that diC4-PI(4,5)P2 has a weaker binding affinity to Arap3-PH1 than diC4-PI(3,4,5)P3.

### 2.4. The PIPs-Binding Ability of Arap3-PH1 Domain Is Required for Arap3 to Inhibit Breast Cancer Cell Invasion In Vitro

**Figure 4:**
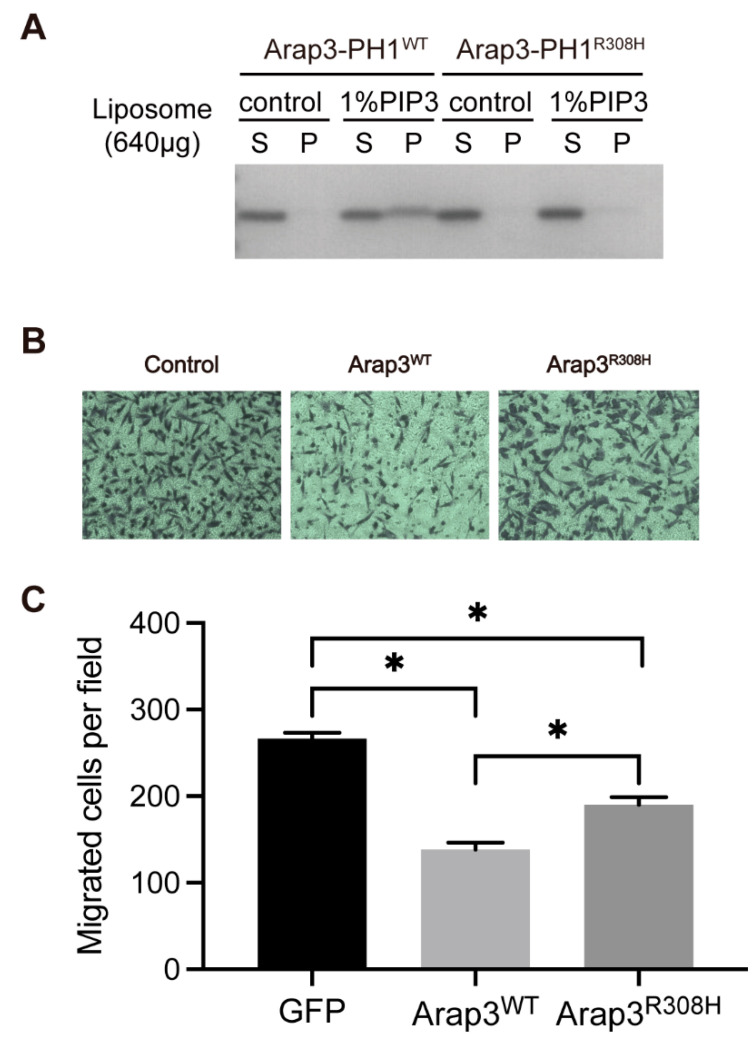
The R308H mutation within the Arap3-PH1 domain abolishes its binding to PI(3,4,5)P3 lipid, and impairs the capacity of Arap3 to inhibit breast cancer cell invasion. (**A**) Liposome binding assays of the Arap3-PH1^R308H^ mutant with liposomes composed of 99% PC and 1% PI(3,4,5)P3. Arap3-PH1^WT^ was used as a positive control. (**B**) Transwell migration assays were performed to measure the cell invasion activities of MDA-MB-231 cells transfected with GFP-Arap3^WT^, GFP-Arap3^R308H^ and the GFP control. (**C**) Quantification of cell invasion activities of cells transfected with GFP-Arap3^WT^, GFP-Arap3^R308H^ and the GFP control from the experiment described in (**B**). Data are expressed as mean ± SEM for each group from three independent experiments. * *p* < 0.05, *p* values were calculated by Student’s *t* test.

Previous study showed that Arap3 can inhibit peritoneal dissemination of scirrhous gastric carcinoma cells [18], and it is also involved in breast cancer [19,20]. We surveyed the COSMIC cancer somatic mutation database, and found that a mutation (R308H) which occurred in Arap3 fell into the PH1 domain. Our structural study shows that R308 is a key residue that directly interacts with the head group of PI(3,4,5)P3. The mutation of this residue will interfere with the ability of Arap3-PH1 to bind PI(3,4,5)P3. We then expressed and purified the Arap3-PH1^R308H^ mutant, and tested its PI(3,4,5)P3-binding ability by liposome pull-down and NMR titration experiments. As expected, the introduction of the R308H mutation in the Arap3-PH1 domain completely abolishes its binding to PI(3,4,5)P3 (Figure 4A and Figure S4). The result is in agreement with previous observations that a double point mutation, R307A/R308A in the first PH domain, totally abolished full-length Arap3 binding to PI(3,4,5)P3 [11]. However, our crystal complex structure shows that R307 is not involved in binding PI(3,4,5)P3 and mutation of this residue may be not necessary.

Arap3 can function as a tumor metastasis suppressor and this function is dependent on its Arf and Rho-GAP activities [18,19], since the plasma membrane recruitment mediated by PI(3,4,5)P3 binding is essential for Arap3 to interact with its substrates Arf and RhoA [12]. We then test whether the PIPs binding ability of the PH1 domain is required for Arap3 to inhibit cancer cell invasion. To explore it, we performed transwell migration assay using the MDA-MB-231 human breast cancer cells. Cells were transfected with GFP-tagged full-length wild-type Arap3, Arap3^R308H^ mutant or the GFP control plasmids (Figure S5). The data show that overexpression of wild-type Arap3 can inhibit the invasion and migration of MDA-MB-231 cells compared with the control. The R308H mutation significantly reduced its inhibitory effect on cell invasion (Figure 4). These results suggest that the lipid-binding ability of PH1 domain is required for Arap3 to inhibit breast cancer cell invasion in vitro.

## 3. Discussion

Arap3 is a PI3K effector, and PI(3,4,5)P3 binding is crucial for its function in cells. In this study, our data demonstrated that the PH1 domain of Arap3 alone can bind PI(3,4,5)P3 efficiently. Our crystal structure revealed that the PI(3,4,5)P3 head group binds specifically at the canonical PIP-binding site on the Arap3-PH1 domain surface. The 1-, 3- and 4-phosphate groups interact directly with the protein. However, the 5-phosphate group is not contacted. This correlates well with the previous observation that Arap3 also preferentially binds PI(3,4)P2 [11]. Moreover, a recent study has shown that local PI(3,4)P2 synthesis can recruit Arap3 to focal adhesions (FAs) and promote FAs turnover [29]. Our data showed that Arap3-PH1 can also bind PI(4,5)P2, which exhibits a weaker affinity than PI(3,4,5)P3. NMR data indicated that diC4-PI(4,5)P2 binds more peripherally than diC4-PI(3,4,5)P3.

It should be noted that our result is in controversy with a previous study reporting that Arap3-PH1 domain alone is unable to bind PIPs [23]. The possible reason for such a discrepancy may due to the different PH1 domain boundaries we used. In the previous study, the PH1 domain was expressed from residue L290 to R394 in Cos7 cell lysis. However, our structure data suggested that residues preceding L290, including P288 and L289, interact with the PH1 domain core and may contribute to the structural integrity and stability of the protein (Figure S6). We, therefore, guess that the Arap3-PH1 construct starting from L290 may not fold correctly; however, this should be further studied.

In cells, the recognition of Arap3-PH1 with PIPs takes place at PM, which mediates the PM recruitment of Arap3 [11,12]. Recent studies suggested that clustering of PIP molecules as well as the lipid composition play key roles in the interactions of a PH domain to a membrane [30,31,32,33,34,35]. How Arap3-PH1 interacts with PIPs-containing membrane should be further investigated, using model membrane systems such as lipid nanodiscs [36,37,38,39]. Moreover, given that Arap3 is a large multi-domain protein, the PIPs-binding ability of its PH1 domain may be regulated by other domains or regions of the protein, which is largely unknown and should also be further investigated.

## 4. Materials and Methods

### 4.1. Plasmid Constructions

The DNA fragments encoding Arap3-PH1 domain (residues 284–385) were cloned into a pET22b (Novagen, Darmstadt, Germany) vector with a C-terminal 6 × His-tag. The expression plasmid encoding the full-length Arap3 with GFP-tagged was constructed as previously described [40]. Mutants were generated by PCR mediated site-directed mutagenesis. All the constructs were verified by DNA sequencing.

### 4.2. Protein Expression and Purification

All the Arap3-PH1 domain constructs with 6×His-tag were transformed into *Escherichia coli* BL21 (DE3) cells (Novagen). Cells were cultured in LB medium at 37 °C up to an OD 600 of 0.8–1.0, and then induced with 0.5 mM IPTG at 16 °C for 24 h. For NMR study, the isotopically labeled Arap3-PH1 proteins were expressed in cells grown in M9 medium containing ^15^N-NH_4_Cl (0.5 g/L) and/or ^13^C-glucose (4 g/L) as the sole nitrogen source and/or carbon source. The ^15^N-NH_4_Cl and ^13^C-glucose were purchased from Cambridge Isotope Laboratories, Inc. All His-tagged proteins were purified by nickel-nitrilotriacetic acid affinity chromatography after lysing cells by sonication in 20 mM Tris/HCl (pH 8.0), 0.5 M NaCl. The Arap3-PH1 proteins were eluted in a 20 mM Tris/HCl (pH 8.0), 0.5 M NaCl with 500 mM imidazole. All proteins were further purified using a Superdex 75 column (GE Healthcare, Piscataway, NJ, USA) in a buffer of 20 mM Tris/HCl (pH 7.5), 200 mM NaCl, 0.5 mM EDTA, 5 mM β-ME. The purity of proteins was confirmed by SDS–PAGE. Protein concentrations were estimated with absorbance spectroscopy, using the molar absorption coefficient.

### 4.3. Liposome Preparation and Liposome Pull-Down Assay

PC (phosphocholine), PI (phosphatidylinositol), PI(4,5)P2 (Phosphatidylinositol 4,5-Bisphosphate) and PI(3,4,5)P3 (Phosphatidylinositol 3,4,5-Trisphosphate) were purchased from Avanti Polar Lipids, Inc. The liposomes containing 2% PI/PI(4,5)P2/PI(3,4,5)P3, 98% PC and 1% PI(3,4,5)P3, 99% PC were prepared for the binding assay. All of the lipids used were dissolved in chloroform and mixed at the molar ratios as described above in glass tubes. The chloroform was dried using nitrogen gas to form a thin film and then further dried under vacuum over 6 h. The dry thin film was hydrated at room temperature in a buffer containing 20 mM Tris/HCl (pH 7.5), and 100 mM NaCl. The suspension was subjected to freeze–thaw cycles in liquid nitrogen and a room temperature water bath for nine cycles, and then sized by an extruder (Avanti Polar Lipids, Birmingham, AL, USA) using a 100 nm polycarbonate filter [41].

Before the experiments, the Arap3-PH1 domain proteins and mutants were dissolved in the same buffer [20 mM Tris/HCl (pH 7.5), 100 mM NaCl] and ultracentrifuged at 70,000× *g*/min for 30 min to remove the precipitate. For the liposome pull-down assay, 20 μg proteins were incubated with of 640 μg liposomes at 4 °C for 30 min, with a total volume of 100 μL. Then, the liposomes were pelleted by centrifugation at 70,000× *g*/min in a Beckman Optima MAX-XP ultracentrifuge for 30 min at 4 °C. The supernatant was removed to determine the free proteins, and the pellets were washed twice with the buffer and then dissolved in 100 μL of SDS/PAGE loading buffer. The supernatant and pellet fractions were subjected to SDS/PAGE and stained by Coomassie Blue.

### 4.4. Surface Plasmon Resonance (SPR)

SPR measurements were conducted in a buffer containing 20 mM HEPES/NaOH (pH 6.8), 100 mM NaCl using a Biacore T200 Instrument (GE Healthcare). A total of 10 μg/mL Arap3-PH1 domain proteins were captured onto the surface of sensor chip CM5 (GE Healthcare) until the response unit (RU) value increased to ∼2500. DiC4-PI(3,4,5)P3 (Echelon, Salt Lake City, UT, USA) were diluted from 75 μM to 1.172 μM as 1:2 dilution series and diC4-PI(4,5)P2 (Echelon, USA) were diluted from 600 μM to 4.688 μM. They were consecutively injected onto the captured Arap3-PH1 proteins. Washing of the flow system using the 20 mM HEPES/NaOH (pH 6.8), 100 mM NaCl for 180 s was then performed following each run. Binding data were analyzed and fitted using a one-site binding model.

### 4.5. Crystallization and Structure

Arap3-PH1 was concentrated to ~9 mg/mL in the buffer of 10 mM Tris/HCl (pH 7.2), 100 mM NaCl, 0.5 mM EDTA and the diC4-PI(3,4,5)P3 was dissolved in the same buffer. Crystals of apo-form Arap3-PH1 domain were crystallized using the sitting drop vapor diffusion method with a one-to-one admixture of the protein and a well solution consisting of 0.05 M calcium chloride dihydrate, 0.1 M BIS-TRIS (pH 6.5), 30% *v/v* polyethylene glycol monomethyl ether 550. Arap3-PH1 was mixed with diC4-PI(3,4,5)P3 at a 1:1.2 molar ratio and crystallized using sitting drop vapor diffusion method at 18 °C by mixing the complex with equilibration solution (0.1 M ammonium sulfate, 0.1 M sodium acetate trihydrate (pH 4.6), 30% *v/v* polyethylene glycol 400) in a one-to-one ratio. All the crystals were transferred to cryoprotectant (dehydration treatment in reservoir solutions containing 15% glycerol) and flash-cooled with liquid nitrogen.

### 4.6. Data Collection and Structure Determination

X-ray diffraction data were collected at 100 K in a liquid nitrogen stream using beamline BL18U at the Shanghai Synchrotron Radiation Facility (SSRF). The data were processed with HKL2000 [42] and programs in the CCP4 suite [43]. The structure of the Arap3-PH1 domain (PDB code: 7YIR) and Arap3-PH1 complexed with diC4-PI(3,4,5)P3 (PDB code: 7YIS) were solved by molecular replacement with co-ordinates from PDB entry 1UPR/1FAO and the program PHASER [44]. All the structural models were subsequently refined by programs REFMAC5 [45], PHENIX [46], and COOT [47]. Crystallographic parameters are listed in Table 1. All structure figures were prepared with PyMOL. The transition state complex interface was calculated in PDBePISA.

### 4.7. Nuclear Magnetic Resonance (NMR) Experiments

All NMR experiments were carried out at 293 K on a Bruker Avance 600 MHz NMR spectrometer equipped with a CryoProbe. All NMR data were processed in NMRPipe [48] and analyzed using Sparky (Goddard and Kneller, University of California, San Francisco). For backbone assignments, 0.75 mM ^15^N,^13^C-labeled Arap3-PH1 protein was dissolved in 50 mM phosphate buffer (pH 6.5) with 50 mM NaCl, 1 mM DTT in 90% H_2_O/10% D_2_O. The following spectra were recorded: 2D ^1^H-^15^N HSQC, 3D-HNCO, HN(CA)CO, HNCA, HN(CO)CA, CBCANH and CBCA(CO)NH.

For NMR titration experiments, the ^15^N-labeled apo Arap3-PH1 and mutants were prepared in NMR buffer: 20 mM Tris/HCl (pH 7.2), 100 mM NaCl, 0.5 mM EDTA, 1 mM DTT in 90% H_2_O/10% D_2_O at a concentration of 100 or 50 μM. Water-soluble lipid analogs diC4-PI(3,4,5)P3 (10 mM) and diC4-PI(4,5)P2 (15 mM) were dissolved into the same NMR buffer to make stock solutions, and the pH was adjusted to pH 7.2 using NaOH. A series of ^1^H-^15^N HSQC spectra were recorded as the titrant gradually titrated into the protein solutions. The weighted chemical shift perturbations (CSPs) for ^15^N and ^1^HN resonances were calculated as follows: Δδ = [(Δδ_HN_)^2^ + (0.17Δδ_N_)^2^]^1/2^.

### 4.8. Cell Cultures, Transfection

MDA-MB-231 cells were maintained in Dulbecco’s modified Eagle’s medium supplemented with 10% (*v/v*) fetal calf serum and penicillin/streptomycin. Cells were transfected using Lipofectamine™ 3000 (Invitrogen, ﻿Carlsbad, CA, USA) reagent according to the manufacturers’ protocols.

### 4.9. Western Blotting and Antibodies

For immunoblotting analyses, MDA-MB-231 cells were lysed in RIPA lysis buffer supplemented with protease and phosphatase inhibitors (Roche Applied Science, Manheim, Germany). Immunoblotting was performed as described previously [40]. Primary antibodies used were anti-GFP tag mAb (Cell Signaling, Danvers, MA, USA), anti-β actin mAb (Cell Signaling, Danvers, MA, USA).

### 4.10. Matrigel Invasion

Before the experiments, MDA-MB-231 cells were transfected with Arap3^WT^, Arap3^R308H^ and empty control, respectively. The expression levels of proteins were verified by immunoblotting. Matrigel invasion assay was performed with Transwell membrane filter inserts (8 mm pore size, Corning costar) by using EGF (10 ng/mL, Sigma-Aldrich, Saint-Louis, MO, USA), as described [49]. Forty-eight hours after transfection, 1 × 10^5^ cells were seeded on the upper wells. After incubation for 12 h, cells were fixed in 4% paraformaldehyde, and the number of cells that migrated out to the lower surface of the membranes was scored by staining with 1% crystal violet. Data were collected from three independent experiments.

## 5. Conclusions

In the present work, we have clarified the phosphoinositide recognition mechanism of Arap3 by biochemical and structural methods. Our data showed that the Arap3-PH1 domain is capable of binding PI(3,4,5)P3 and also PI(4,5)P2, albeit with lower affinity. Crystal structure and NMR titration analysis revealed the structural basis for the specific phosphoinositide recognition by Arap3-PH1. Furthermore, cell-based function analysis using a cancer-associated mutant R308H demonstrated that the lipid-binding ability of the PH1 domain is required for Arap3 to inhibit cancer cell invasion in vitro.

## Figures and Tables

**Table 1:** Crystallographic data collection and refinement statistics.

	Arap3-PH1	Arap3-PH1/diC4-PI(3,4,5)P3 Complex
Data Collection		
Wavelength (Å)	0.9798	0.9798
Space group	P6_1_22	P6_1_22
Cell dimension		
a, b, c (Å)	102.63, 102.63, 45.51	74.15, 74.15, 109.85
α, β, γ (°)	90, 90, 120	90, 90, 120
Resolution * (Å)	50.00–2.10 (2.14–2.10)	50.00–3.30 (3.36–3.30)
Rmerge (%)	11.3 (35.7)	19.6 (48.7)
I/σI	20.5 (7.0)	16.0 (5.0)
Completeness (%)	99.4 (100.0)	100.0 (100.0)
Redundancy	8.9 (8.4)	9.6 (9.5)
Refinement		
No. reflections used/free	8150/424	2633/251
Resolution range (Å)	33.60–2.10	32.11–3.30
*R*_work_/*R*_free_ (%)	18.45/23.00	22.44/27.6
R.m.s. deviations		
Bonds lengths (Å)	0.009	0.004
Bond angles (°)	0.976	0.644
*B*-factors (Å^2^)		
Protein	37.28	57.14
Non-protein	37.04	61.53
No. atoms		
Protein	799	795
Non-protein	53	43
Ramachandran plot		
Favored/allowed/outlier (%)	98.97/1.03/0	94.79/5.21/0

* Statistics for the highest resolution shell is shown in parenthesis.

## Data Availability

Structure data are deposited in the Protein Data Bank with the access code 7YIR and 7YIS.

## Supplementary Materials

The following supporting information can be downloaded at: https://www.mdpi.com/article/10.3390/ijms24021125/s1.
