# Supplementary material for: Structural Insights Uncover the Specific Phosphoinositide Recognition by the PH1 Domain of Arap3
Source: Int J Mol Sci. 2023 Jan 6;24(2):1125. doi: 10.3390/ijms24021125 (PMC9865853; doi:10.3390/ijms24021125)
Supplement: Supplementary file 1 [file ijms-24-01125-s001.zip › ijms-1961438-supplementary.pdf]

Structural insights uncover the specific phosphoinositide recognition by the PH1 domain of Arap3

Youjia Zhang[1, 2, #], Liang Ge[1, #], Li Xu[3], Yongrui Liu[2], Jiarong Wang[1], Chongxu Liu[1, ‡], Hongxin Zhao[1], Lei Xing[1], Junfeng Wang[1,2,4,*] and Bo Wu[1,*]

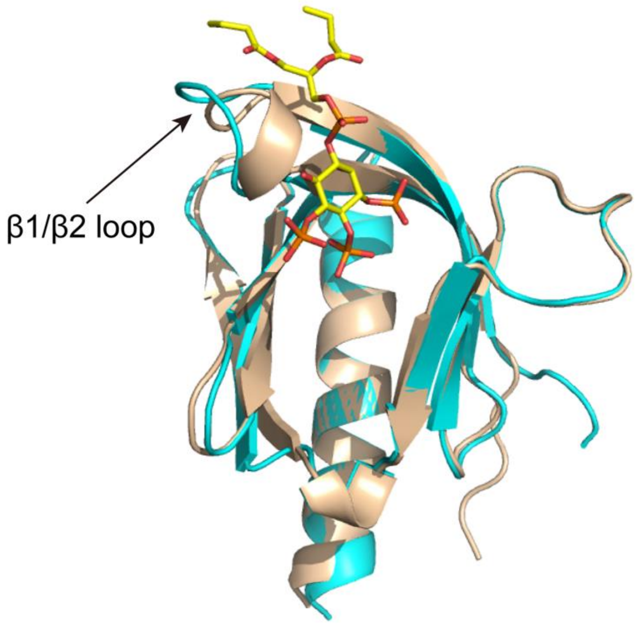

Figure S1. Comparison of the overall structure between unliganded (wheat) and diC4-PI(3,4,5)P3-bound Arap3-PH1 domain (turquoise).

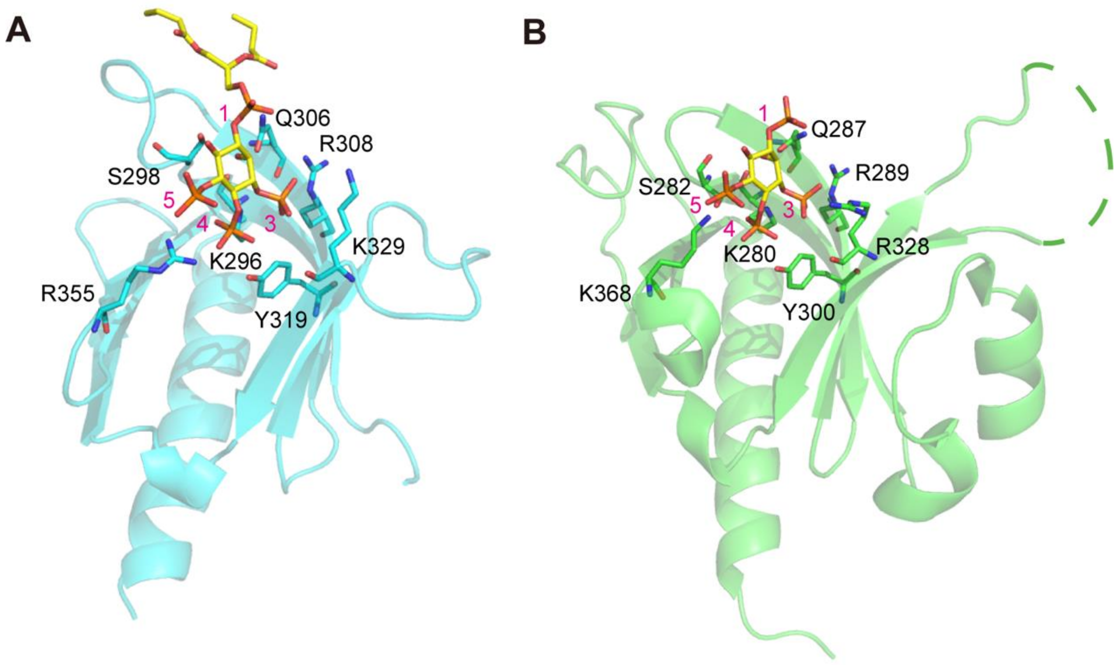

Figure S2. Comparison of the PI(3,4,5)P3-binding sites in Arap3-PH1 and P-Rex1 PH domain. (A) The cartoon presentation of the structure of Arap3-PH1 complexed with diC4-PI(3,4,5)P3. The lipid-binding site residues are depicted as sticks and labeled. (B) The cartoon presentation of the structure of P-Rex1 PH domain in complex with Ins(1,3,4,5)P4, a soluble analog of PI(3,4,5)P3 (PDB code: 5D3X). The similar residues responsible for lipid-binding are depicted as sticks and labeled.

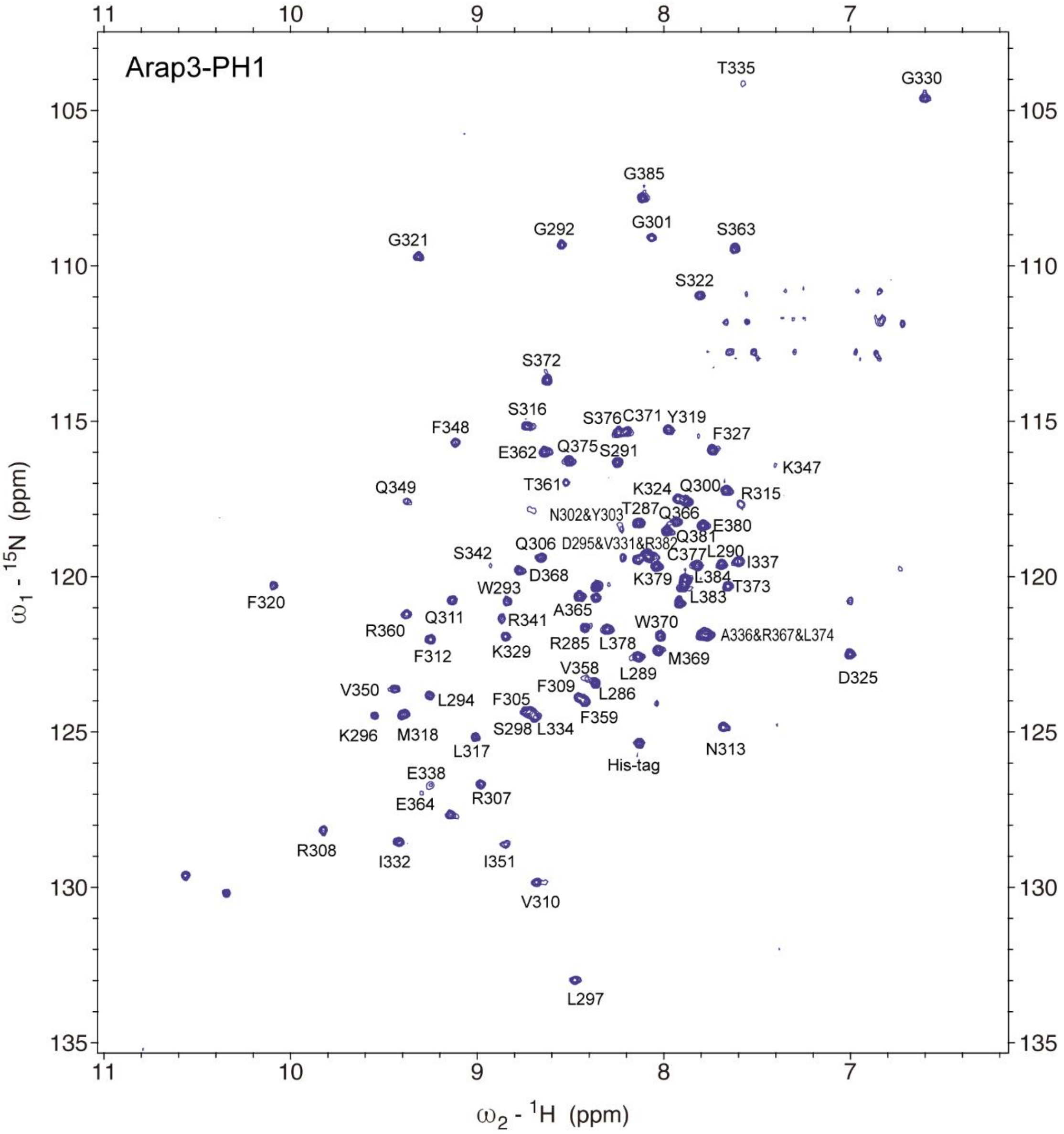

Figure S3. Backbone assignment of Arap3-PH1.

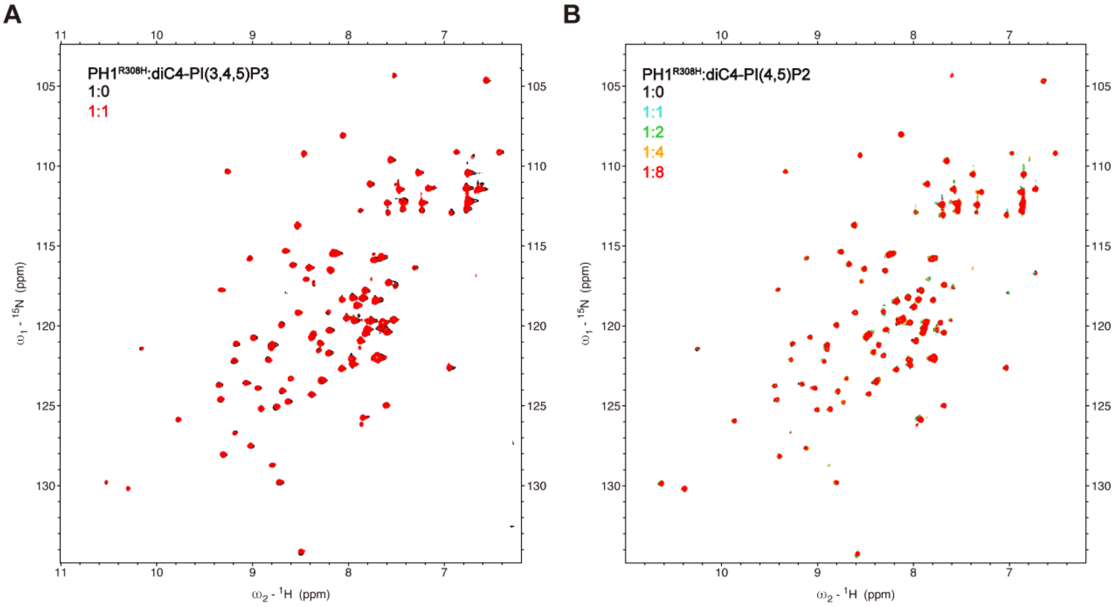

Figure S4. Cancer-associated mutant R308H of Arap3-PH1 abolished its binding to PI(3,4,5)P3/PI(4,5)P2 lipids. (A) Overlay of ^{1}H-^{15}N HSQC spectra of Arap3-PH1^{R308H} in the absence and in the increasing amounts of diC4-PI(3,4,5)P3. The molar ratios of the protein to diC4-PI(3,4,5)P3 are shown in the inset: 1:0 (black) and 1:1 (red). (B) Overlay of ^{1}H-^{15}N HSQC spectra of Arap3-PH1^{R308H} in the absence and in the increasing amounts of diC4-PI(4,5)P2. The molar ratios of the protein to diC4-PI(4,5)P2 are shown in the inset: 1:0 (black), 1:1 (turquoise), 1:2 (lime green), 1:4 (orange) and 1:8 (red).

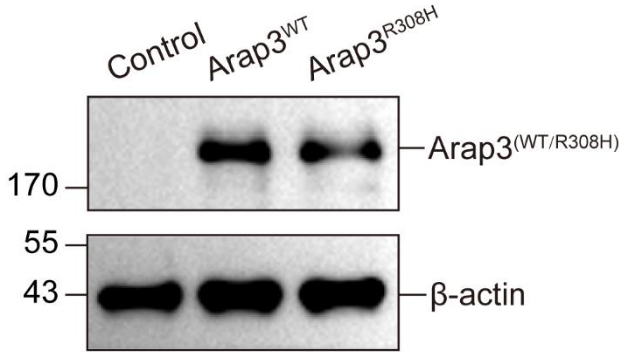

Figure S5. Western blot analysis showing the expression levels of Arap3 and its mutant (anti-GFP), β-actin was used as loading control.

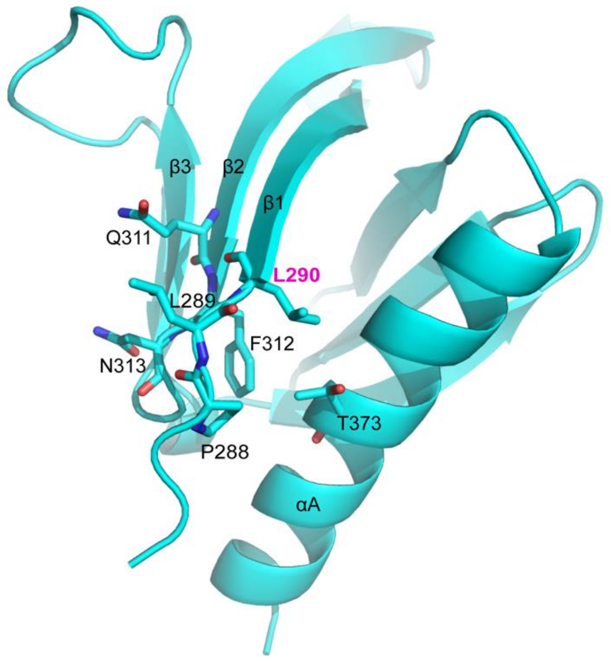

Figure S6. Cartoon representation of Arap3-PH1 domain with the residues P288, L289, L290, Q311, N313 and T373 are shown in stick.
